# Research Progress on the KMT2A-AFF3 Fusion Gene in Childhood Acute Lymphoblastic Leukemia: Mechanisms, Clinical Implications, and Therapeutic Strategies

**DOI:** 10.3390/cimb47120988

**Published:** 2025-11-26

**Authors:** Yawei Zhang, Juan Liang

**Affiliations:** Children’s Hospital, Zhejiang University School of Medicine, National Clinical Research Center for Child Health, Hangzhou 310052, China; zhangyw2017@zju.edu.cn

**Keywords:** KMT2A-AFF3 fusion gene, childhood acute lymphoblastic leukemia, molecular mechanism, targeted therapy, prognosis

## Abstract

KMT2A-rearranged (KMT2A-r) acute lymphoblastic leukemia (ALL), particularly in infants, represents one of the most aggressive pediatric hematological malignancies with a historically dismal prognosis. While KMT2A-AFF1 (t(4;11)) is the most prevalent fusion, a diverse array of partner genes exists, each conferring distinct biological and clinical features. This review focuses on the rare but clinically significant KMT2A-AFF3 subtype, which arises from the t(2;11)(q11.2;q23) chromosomal translocation. This review summarizes the molecular pathogenesis driven by the KMT2A-AFF3 fusion oncoprotein, which functions as an aberrant transcriptional complex. This complex hijacks essential epigenetic machinery, including the recruitment of DOT1L and interaction with Menin, leading to pathogenic histone modifications (e.g., H3K79 hypermethylation) and the subsequent upregulation of critical target genes, notably the HOXA cluster and MEIS1, thereby enforcing a B-lymphoid differentiation arrest at the pro-B/pre-B stage. Clinically, KMT2A-AFF3 ALL is characterized by high-risk features, including infant onset, hyperleukocytosis, central nervous system (CNS) involvement, and a distinct CD10-negative immunophenotype. This review highlights the evidence defining its poor prognosis, which is primarily driven by profound chemoresistance to conventional therapies, including glucocorticoids. Finally, we discuss the rapidly evolving therapeutic landscape, detailing the limitations of standard intensive chemotherapy and the immense promise of novel targeted strategies, such as Menin inhibitors (e.g., Revumenib), DOT1L inhibitors, and immunotherapies (e.g., CAR-T cells, Blinatumomab), which hold the potential to revolutionize outcomes for this high-risk leukemia subtype.

## 1. Introduction

Acute lymphoblastic leukemia (ALL) is the most prevalent malignancy in children, with cure rates now approaching 90% in many developed countries due to risk-stratified chemotherapy protocols [1]. However, specific genetic subtypes continue to confer a high risk of relapse and treatment failure. Among the most challenging of these are leukemias driven by rearrangements of the KMT2A gene (KMT2A-r), located at chromosome 11q23 [2]. KMT2A-r ALL is notoriously aggressive and is the most common genetic abnormality in infant ALL (patients < 1 year of age), where it accounts for 70–80% of cases and is as-sociated with a dismal prognosis, characterized by hyperleukocytosis, early central nervous system (CNS) involvement, and profound resistance to conventional chemotherapy [3].

The KMT2A gene, which encodes a critical histone H3 lysine 4 (H3K4) methyl-transferase, is highly promiscuous and has been documented to fuse with over 100 different partner genes [4]. This diversity is not trivial; the specific fusion partner is a key determinant of the leukemia’s phenotype and clinical behavior. For instance, the KMT2A-AFF1 fusion, arising from the t(4;11)(q21;q23) translocation, is the most common KMT2A rearrangement in both pediatric and adult ALL and is associated with a particularly poor outcome [5]. Other partners, such as MLLT3 (t(9;11)(p22;q23)), are more common in acute myeloid leukemia (AML) but are also found in ALL, presenting different co-mutation landscapes and potential therapeutic vulnerabilities [6]. This prognostic heterogeneity underscores the necessity of characterizing the specific biology driven by each unique KMT2A fusion.

Within this complex landscape, the KMT2A-AFF3 fusion gene, generated by the rare t(2;11)(q11.2;q23) translocation, represents a poorly understood but critical subtype [7]. AFF3 (also known as LAF4) is a member of the AFF (AF4/FMR2) family of transcription factors, which also includes AFF1 (AF4) [8]. While being much rarer than its KMT2A-AFF1 counterpart, KMT2A-AFF3 has been reported in several cases of high-risk infant and pediatric B-cell ALL (B-ALL), often presenting with the same aggressive clinical features, including chemotherapy resistance [7]. Given its rarity, the specific molecular mechanisms, prognostic impact, and optimal therapeutic strategies for KMT2A-AFF3 ALL remain ill-defined, with data often extrapolated from pan-KMT2A-r studies.

This review aims to synthesize the current, albeit limited, state of knowledge specifically focused on KMT2A-AFF3 ALL. We will dissect its molecular characteristics, delineate its unique pathogenic mechanisms involving epigenetic hijacking, summarize its clinical and diagnostic features, and discuss its profound prognostic implications. Finally, we will explore the rapidly advancing therapeutic landscape, highlighting the promise of novel targeted therapies that may provide new hope for patients with this aggressive leukemia subtype. The information synthesized in this review is derived from a search of published literature in databases such as PubMed/MEDLINE and Web of Science using the search terms: ‘KMT2A-AFF3’, ‘t(2;11)(q11.2;q23)’, ‘MLL-LAF4’, ‘MLL-AFF3’, and ‘childhood KMT2A-rearranged ALL’. In addition, we reviewed registered trial data from ClinicalTrials.gov.

## 2. Molecular Characterization

### 2.1. Structure and Function of the KMT2A Gene

The KMT2A (Lysine Methyltransferase 2A) gene, formerly known as MLL (Mixed-Lineage Leukemia), is located at chromosome 11q23. It encodes a large, multi-domain protein that functions as a critical Set1-like histone methyltransferase (HMTase) [9]. The primary role of wild-type KMT2A is to catalyze the trimethylation of histone H3 lysine 4 (H3K4me3), an epigenetic mark robustly associated with active gene promoters. This enzymatic activity is essential for maintaining the transcriptional program of developmental genes, most notably the HOXA cluster genes, which are indispensable for the proper differentiation and self-renewal of hematopoietic stem cells (HSCs) [10]. Structurally, the KMT2A protein contains several functional domains, including AT-hooks for DNA binding, a CXXC domain that binds to unmethylated CpG islands, multiple PHD fingers, and the catalytically active SET domain at its C-terminus. In the context of leukemogenic translocations, the breakpoint in KMT2A invariably occurs within an 8.3 kb region known as the breakpoint cluster region (BCR) (a specific region within the KMT2A gene, not to be confused with the BCR gene on chromosome 22 involved in CML), which separates the N-terminal portion (containing the CXXC domain) from the C-terminal portion (containing the SET domain) [2].

### 2.2. Structure and Function of the AFF3 Gene

The AFF3 (AFF family member 3) gene, located at chromosome 2q11.2, is also known as LAF4. It belongs to the AFF (AF4/FMR2) family of transcription factors, which also includes AFF1 (AF4), AFF2 (FMR2), and AFF4 (AF5q31) [8]. AFF proteins act as crucial scaffolding components within large transcriptional regulatory complexes. Specifically, they are recognized as core components of the Super Elongation Complex (SEC), a multiprotein assembly that includes kinases (such as CDK9/Cyclin T) and other factors (like ELL, AF9, and ENL) [11]. The primary function of the SEC is to phosphorylate the C-terminal domain (CTD) of RNA Polymerase II (Pol II) and suppress its transient pausing, thereby dramatically enhancing the rate and processivity of transcriptional elongation [12]. The AFF3 protein itself contains domains that facilitate these extensive protein–protein interactions, positioning it as a key regulator of gene expression, particularly for genes requiring rapid and high-level transcription.

### 2.3. Formation of the Fusion Gene: t(2;11)(q11.2;q23)

The KMT2A-AFF3 oncoprotein is the product of a rare chromosomal translocation, t(2;11)(q11.2;q23) [7]. This translocation fuses the 5′ region of the KMT2A gene from 11q23 with the 3′ region of the AFF3 gene from 2q11.2. This event results in an in-frame chimeric transcript that, when translated, creates the KMT2A-AFF3 fusion protein. While exceedingly rare compared to the t(4;11) (KMT2A-AFF1) translocation, this specific t(2;11) event has been recurrently identified in pediatric and infant B-ALL cases, often associated with high-risk clinical presentations. In some reported cases, the fusion has been shown to be part of more complex chromosomal rearrangements involving the KMT2A and AFF3 loci, suggesting significant genomic instability [7].

### 2.4. Domain Structure and Biochemical Properties of the KMT2A-AFF3 Fusion Protein

**Figure 1:**
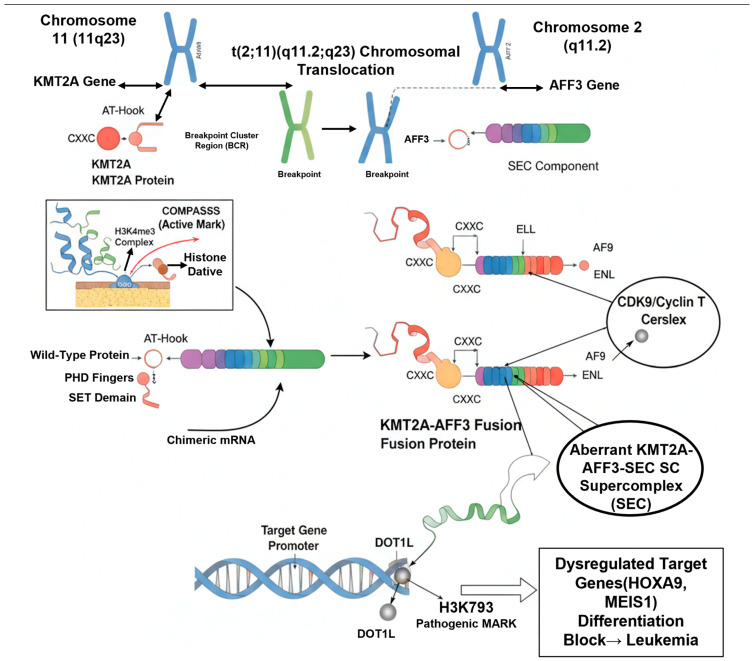
Formation and Transcriptional Reprogramming by the KMT2A-AFF3 Fusion Protein.

The resulting KMT2A-AFF3 fusion protein represents a classic example of a pathogenic gain-of-function oncoprotein. Its structure dictates its leukemogenic mechanism. The fusion protein retains the N-terminal domains of KMT2A, most importantly the CXXC domain, which provides the ability to bind target DNA at CpG islands. However, it loses the C-terminal KMT2A SET domain, and thus, its native H3K4 methyltransferase activity is abolished [9]. In its place, the fusion protein gains the C-terminal portion of AFF3. This AFF3 domain functions as a powerful recruitment platform, effectively “hijacking” the transcriptional elongation machinery, including the SEC [9]. This aberrant chimeric complex is thus mistargeted to the normal KMT2A target gene promoters (e.g., the HOXA cluster). Once bound, the AFF3 component recruits complexes containing the enzyme DOT1L, the sole H3K79 methyltransferase. This leads to a pathogenic shift in histone modifications: the loss of H3K4me3 (due to the missing SET domain) and a gain of aberrant H3K79 hypermethylation at the target loci, driving persistent and uncontrolled high-level transcription of leukemogenic genes and inducing a block in hematopoietic differentiation [13], as shown in Figure 1.

## 3. Pathogenic Mechanisms

The leukemogenicity of the KMT2A-AFF3 fusion is not attributed to a single aberrant function but rather to a multi-step process of transcriptional and epigenetic hijacking. This process culminates in the establishment of a robust, self-reinforcing oncogenic program that blocks normal hematopoietic differentiation and drives malignant proliferation.

### 3.1. Aberrant Transcriptional Complex Formation

The KMT2A-AFF3 fusion protein acts as a pathogenic scaffold, fundamentally rewiring the cell’s transcriptional apparatus. Wild-type KMT2A is normally a core component of the COMPASS (Complex of Proteins Associated with Set1) complex, which mediates H3K4 trimethylation [9]. In stark contrast, the KMT2A-AFF3 fusion protein acquires a neomorphic (new) function. As detailed in Section 2.4, the fusion protein retains the N-terminal domains of KMT2A, providing the crucial DNA-binding capability to target HOXA cluster genes and other developmental loci. However, it loses the C-terminal SET domain.

In its place, the C-terminal AFF3 moiety provides a potent new interaction surface. AFF3 is a native and essential scaffolding component of the Super Elongation Complex (SEC), a powerful transcriptional assembly whose members include the P-TEFb kinase (CDK9/Cyclin T), ELL (Eleven-nineteen L-cell leukemia), AF9 (MLLT3), and ENL (MLLT1) [11]. By fusing with AFF3, the KMT2A N-terminus effectively commandeers the entire SEC, creating a chimeric “KMT2A-AFF3-SEC” super complex. This aberrant complex is then mistargeted a masse to KMT2A’s native genomic binding sites, setting the stage for profound epigenetic dysregulation [12]. This mechanism of SEC hijacking is a common pathogenic theme for KMT2A fusions; indeed, AFF1 (the partner in t(4;11)) and AFF4 (a partner in t(X;11)) are also members of the same AFF family and hijack the SEC in an analogous manner [11].

### 3.2. Epigenetic Reprogramming: The Role of DOT1L and Menin

The primary pathogenic mechanism of KMT2A-r oncoproteins, including KMT2A-AFF3, is the aberrant recruitment of epigenetic modifiers. Two cofactors are absolutely essential for this process:

DOT1L Hijacking and H3K79 Hypermethylation: The SEC, now part of the KMT2A-AFF3 complex, potently recruits the enzyme DOT1L (DOT1-like), which is the sole histone methyltransferase responsible for H3K79 methylation [14]. This recruitment leads to a dramatic and pathogenic shift in local chromatin structure: DOT1L is aberrantly targeted to the promoters and gene bodies of KMT2A-AFF3 target genes (e.g., HOXA9), resulting in focal hypermethylation of H3K79 (specifically H3K79me2/3) [13]. These H3K79me2/3 marks are powerful “on” signals that facilitate transcriptional elongation. This “H3K79 hypermethylation signature” is a hallmark of KMT2A-r leukemias and is directly responsible for the high-level, sustained expression of the leukemogenic gene program [15].

Menin as an Essential Anchor: The protein Menin (encoded by the MEN1 gene) binds directly to the retained N-terminal portion of KMT2A within the fusion protein [16]. This interaction is indispensable for leukemia. Menin acts as a critical tether or anchor, stabilizing the binding of the entire KMT2A-AFF3 oncoprotein complex to chromatin at its target loci. Without Menin, the fusion protein cannot efficiently bind to chromatin, and the downstream recruitment of DOT1L and the SEC fails. This critical dependency on the Menin-KMT2A interaction has been identified as a key therapeutic vulnerability across nearly all KMT2A-r leukemias [17].

### 3.3. Downstream Effects: Target Gene Dysregulation and Differentiation Arrest

The consequence of this epigenetic reprogramming is the massive and sustained dysregulation of a specific set of downstream target genes. The most critical and consistently upregulated targets in KMT2A-r leukemias are the HOXA cluster genes, particularly HOXA7, HOXA9, and HOXA10, along with their key cofactor, MEIS1 [9]. These genes are master regulators of hematopoietic stem cell (HSC) self-renewal and proliferation. In normal hematopoiesis, their expression is tightly controlled and rapidly silenced as progenitor cells commit to lymphoid differentiation [18].

The KMT2A-AFF3 oncoprotein—via the mechanisms described above—prevents this crucial silencing. It forces the persistent, high-level expression of this HOXA/MEIS1 program in B-cell progenitors. This aberrant HOXA/MEIS1 activity is the direct effector of the leukemic phenotype. It imposes a “stem-cell-like” transcriptional program onto the committed progenitor cell, short-circuiting the normal developmental pathway and causing a “differentiation arrest” [18]. This arrest, typically occurring at the pro-B or pre-B cell stage, is the physical manifestation of the disease and is consistent with the aggressive, CD10-negative immunophenotype often observed in these patients. Beyond the HOXA axis, this oncogenic program also drives the expression of other critical survival genes, such as the anti-apoptotic factor BCL-2 and the stem cell marker FLT3 (FMS-like tyrosine kinase 3), both of which contribute to the leukemia’s survival and chemoresistance [19].

### 3.4. Activation of Downstream Signaling Pathways

While the HOXA/MEIS1 axis represents the primary oncogenic driver, the KMT2A-AFF3 fusion establishes a cellular state that fosters the hyperactivation of multiple parallel signaling pathways. These pathways are not just bystanders; they are essential for providing the robust proliferation, survival, and metabolic signals that cooperate with the differentiation block to create a fully malignant phenotype. This creates a feed-forward loop, as the HOXA targets themselves can, in turn, further activate some of these pathways [20]. Key activated pathways are summarized in Table 1.

The activation of these pathways provides critical context for the disease’s aggressiveness. For instance, the hyperactivation of the PI3K/AKT/mTOR pathway is a master regulator of chemoresistance. It not only drives resistance to glucocorticoids (a cornerstone of ALL therapy) by inactivating the glucocorticoid receptor [22], but also provides a powerful pro-survival signal that allows leukemic cells to bypass apoptosis. Furthermore, it fuels the high metabolic and protein synthesis demands (via mTORC1) of rapid proliferation [23].

**Figure 2:**
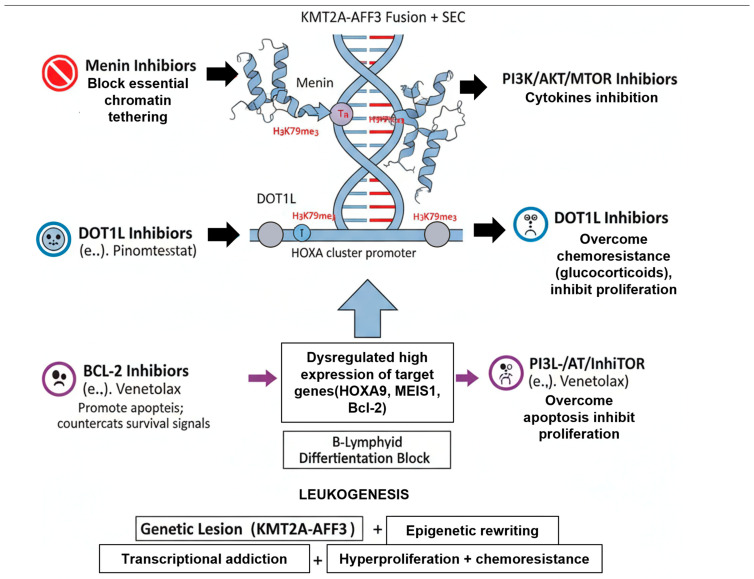
Core Pathogenic Mechanisms and Targeted Therapeutic Interventions in KMT2A-AFF3-Driven Leukemia.

Similarly, the RAS/MAPK pathway is central to the “second hit” model of leukemogenesis. The KMT2A-AFF3 fusion is the initiating lesion that establishes the differentiation block. However, it often requires a cooperating proliferative signal to drive rapid, full-blown leukemia. Activating mutations in NRAS or KRAS are the most common “second hits” that provide this relentless “go” signal, cooperating with the KMT2A-AFF3-driven self-renewal program [24]. The JAK/STAT pathway functions similarly, often being activated in an autocrine fashion by KMT2A-r targets like FLT3 or IL-7R, thereby fueling a constant stream of pro-survival and pro-proliferation signals [19,25], as shown in Figure 2.

## 4. Clinical and Diagnostic Features

The KMT2A-AFF3 fusion gene defines a rare but clinically distinct subgroup of B-cell acute lymphoblastic leukemia (B-ALL) characterized by aggressive clinical features and specific diagnostic markers. Its identification requires a multi-modal approach, integrating clinical presentation, immunophenotyping, and detailed genetic analysis.

### 4.1. Epidemiology and Demographics

KMT2A rearrangements (KMT2A-r) collectively represent a major high-risk subgroup in pediatric ALL. They have a unique age distribution, being disproportionately common in infants (patients < 1 year of age), where they account for 70–80% of all ALL cases [3,26]. In older children, the incidence of KMT2A-r ALL is significantly lower, representing approximately 5–10% of cases.

Within this KMT2A-r category, the KMT2A-AFF3 fusion is exceptionally rare. The vast majority of KMT2A-r B-ALL cases are defined by the t(4;11)(q21;q23) translocation, which generates the KMT2A-AFF1 fusion [4]. KMT2A-AFF3 is one of the numerous “other” KMT2A fusion partners that are now being identified with increasing frequency due to the widespread adoption of advanced molecular techniques like RNA-sequencing. These reports confirm its presentation in the pediatric population, including infants and young children [7], consistent with the typical age distribution of KMT2A-r leukemias.

### 4.2. Clinical Presentation and Laboratory Findings

Patients with KMT2A-AFF3 ALL typically present with a highly aggressive disease phenotype, mirroring the characteristics of other high-risk KMT2A-r leukemias. The clinical picture at diagnosis is often dramatic and indicative of a rapidly proliferating disease.

#### 4.2.1. Hyperleukocytosis

A hallmark of KMT2A-r ALL is an extremely high white blood cell (WBC) count at presentation, often exceeding 100,000/µL and sometimes reaching >500,000/µL [3]. This hyperleukocytosis is a direct consequence of the rapid, uncontrolled proliferation of lymphoblasts driven by the oncoprotein and contributes to immediate complications such as leukocytosis and tumor lysis syndrome.

#### 4.2.2. Central Nervous System (CNS) Involvement

KMT2A-r leukemias, including KMT2A-AFF3, exhibit a strong propensity for early infiltration of the central nervous system (CNS) [3]. The presence of leukemic blasts in the cerebrospinal fluid (CSF) at diagnosis is common and necessitates intensive CNS-directed therapy from the outset.

#### 4.2.3. Organomegaly

Similar to other acute leukemias, significant enlargement of the spleen (splenomegaly) and liver (hepatomegaly), as well as lymphadenopathy, is frequently observed due to widespread leukemic infiltration.

#### 4.2.4. Poor Initial Treatment Response

A key prognostic indicator in pediatric ALL is the response to the initial prednisone/dexamethasone prophase. KMT2A-r leukemias are notoriously resistant to glucocorticoids, often demonstrating a poor prednisone response (PPR) [26]. This initial chemoresistance, linked to mechanisms including PI3K/AKT pathway activation (as discussed in Section 3), immediately classifies these patients into the highest risk categories of contemporary treatment protocols [3,26].

### 4.3. Immunophenotype

The immunophenotypic profile of KMT2A-AFF3 ALL is critical for diagnosis and classification. By flow cytometry, the leukemic blasts consistently display a B-cell lineage (CD19+, cytoplasmic CD79a+, cytoplasmic CD22+) but are arrested at a very early stage of development, corresponding to a pro-B or pre-B (B-I/B-II) stage.

The most characteristic feature is the negative expression of CD10 [27]. This contrasts sharply with the most common forms of pediatric B-ALL, which are CD10-positive (“common ALL”). This CD10-negative phenotype is a classic hallmark of KMT2A-r B-ALL. Furthermore, these blasts often show “lineage infidelity” or “lineage promiscuity” by aberrantly co-expressing myeloid-associated markers, such as CD15, CD33, or CD68 [26]. The blasts are also typically positive for early progenitor markers, including CD34 and TdT (Terminal deoxynucleotidyl transferase) [3,7]. This specific immunophenotype (CD19+, CD10−, often CD34+, with or without myeloid markers) is highly suggestive of a KMT2A rearrangement and mandates immediate genetic investigation.

### 4.4. Cytogenetics and Molecular Diagnostics

A definitive diagnosis of KMT2A-AFF3 ALL requires genetic confirmation. A tiered diagnostic workflow, summarized in Table 2, is essential to first identify the KMT2A rearrangement and then to pinpoint AFF3 as the specific fusion partner.

#### 4.4.1. Conventional Cytogenetics (Karyotyping)

G-banded karyotyping may reveal the causative translocation, t(2;11)(q11.2;q23) [7]. However, this translocation can be subtle or “cryptic” and easily missed. In several reported instances, the fusion resulted not from a simple reciprocal translocation but from a highly complex chromosomal rearrangement involving three or more chromosomes, making interpretation by karyotyping alone impossible [7].

#### 4.4.2. Fluorescence In Situ Hybridization (FISH)

FISH is the standard rapid screening test for KMT2A-r ALL. The test employs a “break-apart” probe set that hybridizes to the KMT2A locus on chromosome 11q23. In normal cells, the probes co-localize, producing a fused signal (e.g., yellow or red-green fusion). In leukemic cells with a KMT2A rearrangement, the probes are pulled apart, resulting in a “split signal” pattern (e.g., one separate red, one separate green, and one residual fusion signal). This test confirms a KMT2A break-apart with high sensitivity but does not identify the partner gene.

#### 4.4.3. Reverse-Transcription PCR (RT-PCR)

This technique uses a forward primer specific to a KMT2A exon (e.g., exon 8, 9, or 10) and a reverse primer specific to an AFF3 exon. The amplification of a product of the expected size confirms the KMT2A-AFF3 chimeric transcript.

#### 4.4.4. Next-Generation Sequencing (NGS)

NGS-based methods, particularly RNA-sequencing (RNA-seq) or targeted transcriptome-based fusion panels, are now the gold standard. They can simultaneously detect all known and novel fusions in an unbiased manner. This is crucial for KMT2A-r, where over 100 different partners exist. RNA-seq will definitively identify the KMT2A-AFF3 fusion, map the exact genomic breakpoints, and simultaneously screen for cooperating “second hit” mutations (e.g., in KRAS, NRAS, PTPN11) that are critical for risk stratification and prognostic assessment [24].

#### 4.4.5. Minimal Residual Disease (MRD) Monitoring

The unique KMT2A-AFF3 fusion junction sequence created by the translocation is a patient-specific tumor marker. After diagnosis, quantitative PCR (qPCR) or, more recently, digital droplet PCR (ddPCR) assays are designed to target this specific junction. These highly sensitive techniques can detect one leukemic cell among 100,000 to 1,000,000 normal cells [27]. This level of MRD monitoring is essential for assessing early treatment response, guiding therapy intensification (such as the decision for HSCT), and detecting impending relapse long before it becomes clinically apparent.

## 5. Prognosis and Risk Stratification

The identification of specific genetic drivers in ALL is the cornerstone of modern risk-adapted therapy. The KMT2A-AFF3 fusion, like most KMT2A rearrangements, is a powerful and independent indicator of a high-risk disease entity, mandating aggressive, upfront therapeutic strategies from the moment of diagnosis.

### 5.1. Prognostic Significance of KMT2A-AFF3

KMT2A rearrangements (KMT2A-r) in pediatric ALL are, as a group, one of the most reliable markers of a poor prognosis, particularly in infants [3,26]. Historical data from large cooperative groups (e.g., Children’s Oncology Group (COG), Berlin-Frankfurt-Münster (BFM) group) consistently show that infants with KMT2A-r ALL have event-free survival (EFS) rates significantly lower than those without this rearrangement, and their prognosis is markedly inferior to that of older children [26].

Due to its extreme rarity, the specific prognosis of KMT2A-AFF3 has not been defined in large cohort studies. Its prognostic assessment must therefore be inferred from case reports and its classification within the broader KMT2A-r “high-risk” category. Case reports describing KMT2A-AFF3 ALL often detail the classic features of high-risk disease: presentation in infancy or early childhood, hyperleukocytosis, and a clinical course marked by aggressive disease and early relapse [7].

It is critical to differentiate it from the most common KMT2A-r fusion, KMT2A-AFF1 (t(4;11)). KMT2A-AFF1 is widely regarded as conferring the most dismal prognosis of all B-ALL subtypes, especially in infants, with a very high rate of early and refractory relapse [3]. While data is limited, KMT2A-AFF3 is similarly categorized as a high-risk lesion, and patients are uniformly channeled into high-risk treatment arms. However, as an area of limited current knowledge, it is not yet clear whether the AFF3 partner confers an identical prognostic risk or if its specific biology results in a subtly different phenotype (e.g., more or less aggressive) compared to the dismal prognosis associated with AFF1. The primary driver of this poor prognosis is not just the fusion itself, but its profound biological consequences: a block in B-cell differentiation, the activation of potent pro-survival signaling pathways (e.g., PI3K/AKT), and an intrinsic, de novo resistance to standard-of-care agents like glucocorticoids [22,23].

### 5.2. Role in Clinical Risk Stratification

Modern ALL treatment protocols utilize a multi-factorial system to stratify patients into risk groups (e.g., standard-risk, intermediate-risk, high-risk, very-high-risk) to appropriately de-escalate or intensify therapy [28]. The presence of KMT2A-AFF3 is a dominant factor in this stratification, overriding most other favorable features.

Genetic Stratification: The detection of a KMT2A rearrangement by FISH or NGS is, by itself, a sufficient criterion to assign a patient to a high-risk (HR) or very-high-risk (VHR) protocol. This genetic finding immediately flags the leukemia as biologically aggressive and chemo resistant. The specific partner, AFF3, confirms the lesion is pathogenic and does not alter this high-risk assignment.

Complicating Clinical Factors: The high-risk status conferred by the KMT2A-AFF3 fusion is often complicated by other classical high-risk features. As discussed in Section 4, these patients frequently present with:

Age: Infancy (<1 year), especially <6 months, is an independent variable of very poor prognosis [26]. WBC Count: Hyperleukocytosis (>50,000/µL or >100,000/µL) at diagnosis is another powerful, independent high-risk factor. A patient presenting with all three—infant age, hyperleukocytosis, and a KMT2A rearrangement—is placed in the highest possible risk category, anticipating a poor response to standard therapy.

Response-Based Stratification (MRD): The single most important prognostic factor in modern ALL therapy is the measurement of Minimal Residual Disease (MRD) [29]. MRD assesses the in vivo chemosensitivity of the leukemic clone. Patients with KMT2A-r ALL, including KMT2A-AFF3, are closely monitored. A rapid and deep MRD response (e.g., <0.01% or undetectable by the end of induction therapy) is a strong favorable sign, even in this high-risk group. Conversely, persistent MRD (e.g., >0.1% or >1% at induction end) is a dire prognostic indicator, confirming the in vivo chemoresistance of the clone and serving as a primary indication for allogeneic hematopoietic stem cell transplantation (HSCT) [30].

### 5.3. Key Prognostic Factors and Challenges

For a patient diagnosed with KMT2A-AFF3 ALL, several key factors are monitored to refine prognosis and guide treatment decisions, as summarized in Table 3. The overarching prognostic challenge is the high propensity for early and aggressive relapse, often occurring on therapy or shortly after its completion. This is driven by the intrinsic resistance of the KMT2A-driven leukemia stem cell (LSC) population, which can survive initial chemotherapy and re-initiate the disease.

## 6. Treatment Strategies

The treatment landscape for KMT2A-AFF3 ALL is defined by its upfront classification as a very-high-risk (VHR) disease. The therapeutic approach is multi-modal, intensive, and increasingly reliant on molecularly targeted and immunotherapeutic agents to overcome the profound intrinsic chemoresistance characteristic of the KMT2A-r subgroup.

### 6.1. Intensive Chemotherapy Backbones

Given their VHR status, patients with KMT2A-AFF3 ALL are ineligible for standard-risk or intermediate-risk protocols. They are immediately assigned to the most intensive arms of national and international clinical trials (e.g., COG VHR arms, AIEOP-BFM high-risk blocks) [28,31]. These protocols are characterized by:

Multi-Agent Induction: An intensified 4- or 5-drug induction regimen, typically including vincristine, a potent glucocorticoid (dexamethasone), L-asparaginase, an anthracycline (e.g., daunorubicin), and sometimes cyclophosphamide.

Early Response Assessment: A poor response to the initial 7-day prednisone prophase is common in KMT2A-r ALL and is a key adverse prognostic factor [26].

Intensive CNS-Directed Therapy: Due to the high propensity for CNS infiltration, therapy includes repeated, high-dose systemic methotrexate (HD-MTX) and frequent triple intrathecal (ITT) chemotherapy (methotrexate, cytarabine, hydrocortisone) throughout all treatment phases [31].

Despite this intensification, conventional chemotherapy alone is often insufficient to cure KMT2A-r ALL. The intrinsic chemoresistance, particularly to glucocorticoids, and the high rate of early relapse underscore the urgent need for consolidative therapies and novel agents [3].

In summary, while intensive multi-agent chemotherapy serves as the necessary backbone, its efficacy is often insufficient on its own due to the profound intrinsic chemoresistance of this leukemia subtype.

### 6.2. Allogeneic Hematopoietic Stem Cell Transplantation (HSCT)

Allogeneic HSCT remains a critical consolidative modality for the highest-risk pediatric ALL. For KMT2A-AFF3 patients, the decision for HSCT is primarily driven by their early treatment response, as measured by MRD [30].

HSCT in first complete remission (CR1) is strongly indicated for KMT2A-r patients who demonstrate a poor MRD response, such as failure to achieve MRD negativity (<0.01%) after induction or early consolidation blocks.

For patients with persistent, high-level MRD (>1%) or those who are refractory to induction, HSCT is often the only curative option, though it is frequently preceded by “bridge” therapies (e.g., Blinatumomab or CAR-T) to first achieve a deep remission [32].

KMT2A-r patients who achieve a rapid and deep MRD-negative remission may be eligible for chemotherapy-only protocols, avoiding the significant long-term morbidity and mortality of HSCT. However, the threshold to recommend HSCT (specifically, the level of persistent MRD that would trigger an HSCT recommendation) is generally much lower for this genetic subgroup compared to standard-risk ALL [32,33].

Ultimately, HSCT remains a critical consolidative tool, with the decision to proceed being almost entirely dependent on the individual patient’s MRD response as a measure of in vivo chemosensitivity.

### 6.3. Targeted Therapies: Exploiting Core Dependencies

The failures of conventional chemotherapy have spurred intensive research into the core biological dependencies of KMT2A-r cells. This has yielded a new class of “epigenetic” drugs that target the oncoprotein’s pathogenic mechanism directly, representing the most significant therapeutic advance in this field.

This is arguably the most promising targeted strategy. As discussed in Section 3, the interaction between the KMT2A N-terminus and the protein Menin is an indispensable anchor for the oncoprotein complex [16]. Small-molecule inhibitors that disrupt this Menin-KMT2A interaction (e.g., Revumenib/SNDX-5613, Ziftomenib/KO-539) have shown remarkable efficacy. These drugs work by evicting the KMT2A-AFF3 complex from chromatin, leading to the rapid shutdown of the HOXA/MEIS1 gene program, induction of hematopoietic differentiation, and potent anti-leukemic activity [17]. Phase I/II clinical trials of Revumenib in heavily pretreated R/R KMT2A-r and NPM1-mutant acute leukemias (both AML and ALL) have demonstrated high response rates and achieved deep, MRD-negative remissions, leading to breakthrough therapy designations [33,34].

This was the first rationally designed epigenetic therapy for KMT2A-r leukemia. These inhibitors (e.g., Pinometostat/EPZ-5676) block the DOT1L enzyme, thereby preventing the aberrant H3K79 hypermethylation that is essential for HOXA gene expression [14]. While showing strong pre-clinical promise, Pinometostat demonstrated only modest single-agent activity in clinical trials, particularly in ALL [35]. Its development has largely been superseded by the more potent Menin inhibitors, though it is still being explored in combination therapies.

The KMT2A-r transcriptional program, via HOXA9, drives high-level expression of the anti-apoptotic protein BCL-2, rendering the cells “primed for apoptosis”. Venetoclax, a potent BCL-2 inhibitor, has shown significant pre-clinical activity against KMT2A-r ALL, especially when combined with chemotherapy (e.g., glucocorticoids) or other targeted agents [36]. It is being actively investigated in clinical trials as a means to overcome glucocorticoid resistance.

Given the hyperactivation of signaling pathways like PI3K/AKT/mTOR and JAK/STAT (Section 3.4), inhibitors targeting these nodes are under investigation. Inhibitors of PI3K (e.g., Idelalisib) or AKT (e.g., Capivasertib) may help re-sensitize KMT2A-r cells to glucocorticoids [23]. Similarly, FLT3 inhibitors (e.g., Gilteritinib) may have a role, as FLT3 is often a downstream target of the KMT2A oncoprotein, even in the absence of a canonical FLT3-ITD mutation [37].

These targeted agents, particularly the Menin inhibitors, represent the most significant therapeutic advance, moving treatment away from non-specific chemotherapy and towards exploiting the core epigenetic dependencies of the disease, as summarized in Table 4.

### 6.4. Immunotherapies

For patients who relapse or are refractory to chemotherapy, immunotherapy has revolutionized outcomes.

Chimeric Antigen Receptor (CAR) T-cell therapy targeting CD19 (e.g., Tisagenlecleucel, Axicabtagene ciloleucel) is a standard-of-care for R/R B-ALL, capable of inducing deep, durable remissions [43]. KMT2A-r patients are frequent candidates for this therapy due to their high relapse rate. However, KMT2A-r ALL poses a unique challenge to CAR-T therapy: CD19-negative relapse via lineage switching. The underlying plasticity of the KMT2A-r cell allows it to “escape” immune pressure by shedding its B-cell (CD19) identity and “switching” to a myeloid phenotype (CD19-negative, CD33/CD123-positive) [7]. This remains a major mechanism of failure for CD19-directed immunotherapy in this specific subgroup.

Blinatumomab is a BiTE molecule that links CD19 on blasts to CD3 on T-cells, inducing T-cell-mediated cytotoxicity. It is approved for R/R ALL and, critically, for the treatment of MRD-positive B-ALL [32,44]. For KMT2A-AFF3 patients who fail to clear MRD with chemotherapy, Blinatumomab serves as a powerful and less-toxic consolidation strategy to deepen remission, often used as a “bridge” to curative HSCT [45].

Inotuzumab Ozogamicin, an ADC targeting CD22, provides another potent immunotherapeutic option, particularly for patients who may have relapsed after CD19-directed therapy or have low/heterogeneous CD19 expression [46].

In summary, immunotherapies such as CAR T-cells and BiTE molecules (Blinatumomab) have become indispensable tools for managing relapsed or refractory disease, although their long-term success in KMT2A-r ALL may be challenged by the unique mechanism of CD19-negative relapse via lineage switching.

## 7. Conclusions and Future Perspectives

The KMT2A-AFF3 fusion gene, generated by the rare t(2;11)(q11.2;q23) translocation, defines a distinct and highly aggressive molecular subtype of pediatric B-cell acute lymphoblastic leukemia (B-ALL). Although its rarity has precluded its characterization in large, independent clinical trials, a clear and consistent picture has emerged from case studies and its classification within the broader, well-defined KMT2A-rearranged (KMT2A-r) VHR-ALL subgroup. This entity is synonymous with a grim prognosis, driven by its typical presentation in infancy, profound chemoresistance, and a high propensity for early and aggressive relapse.

This review has synthesized the current understanding of KMT2A-AFF3 ALL, framing it as a disease of transcriptional and epigenetic dysregulation. The fusion oncoprotein acts as a potent aberrant transcription factor, hijacking the core Menin and DOT1L/AF4-FMR2 (AFF) complexes to execute a pathogenic epigenetic program. This leads to the hallmark H3K79 hypermethylation at key loci, driving the high-level expression of oncogenes like HOXA9, MEIS1, and BCL-2. This single molecular event provides a cogent, unified mechanism explaining the disease’s key features: the pro-B differentiation block (CD10-negative immunophenotype), the rampant proliferation (hyperleukocytosis), and the intrinsic resistance to conventional therapies like glucocorticoids (poor MRD response).

Despite the poor outcomes associated with conventional intensive chemotherapy and HSCT, the therapeutic landscape for KMT2A-AFF3 ALL is on the verge of a paradigm shift. The elucidation of the oncoprotein’s core dependencies has ushered in an era of rationally designed targeted therapies. The most transformative of these are the Menin inhibitors (e.g., Revumenib), which directly disrupt the indispensable KMT2A-Menin interaction, reverse the pathogenic gene signature, and induce differentiation. The remarkable clinical activity of these agents in relapsed/refractory KMT2A-r leukemias provides a powerful new backbone upon which future therapies will be built. It is important to note that while this review focuses on ALL, the fundamental epigenetic mechanisms driven by KMT2A rearrangements—specifically the dependency on Menin and DOT1L—are conserved across hematopoietic lineages. Consequently, critical mechanistic insights and therapeutic rationales are often shared between KMT2A-rearranged ALL and acute myeloid leukemia (AML), justifying the broad applicability of these targeted strategies.

However, significant challenges remain. The plasticity of KMT2A-r blasts poses a major threat to immunotherapy, with high rates of CD19-negative relapse via myeloid lineage switching observed after CAR T-cell therapy [7]. Furthermore, a critical gap in knowledge, highlighted by the fusion’s rarity, is whether the AFF3 partner contributes unique biological properties distinct from the more common AFF1 partner. While both are AFF family members that hijack the SEC, it remains poorly understood whether the specific C-terminal domains of AFF3 mediate different protein–protein interactions (e.g., recruiting distinct chromatin modifiers) or enforce a subtly different transcriptional profile, which could present unique therapeutic vulnerabilities. This remains a key direction for future functional modeling studies.

Future research must proceed along parallel translational and basic science tracks. Clinically, the future lies in intelligent combination therapies. Prospective trials are urgently needed to evaluate the integration of Menin inhibitors into frontline chemotherapy backbones for newly diagnosed patients. We envision that future protocols for newly diagnosed KMT2A-AFF3 ALL will move beyond traditional chemotherapy-centric induction. A potential future standard could involve an initial ‘epigenetic induction’ phase, combining a Menin inhibitor like Revumenib directly with a BCL-2 inhibitor (Venetoclax), aimed at rapidly reversing the core transcriptional program and inducing deep, early MRD negativity. This targeted approach could then be consolidated with less-intensive, response-adapted chemotherapy or immunotherapy, potentially reducing the reliance on high-dose systemic agents and allogeneic HSCT for all patients. Moreover, rational combinations, such as Menin inhibitors with BCL-2 inhibitors (Venetoclax) or PI3K/AKT inhibitors, hold immense promise for creating deep, synergistic, and durable remissions [34,36]. Strategies to overcome immunotherapy escape, such as dual-antigen targeting CAR-T cells (e.g., CD19/CD22 or CD19/CD123), will also be essential. From a basic science perspective, the field requires the development of specific KMT2A-AFF3 in vivo and in vitro models (e.g., patient-derived xenografts, isogenic cell lines) to definitively dissect the partner-specific functions of AFF3 and to serve as a platform for pre-clinically validating these novel combination therapies.

Answering this clinically relevant question will be critical for personalizing therapy. Potential early predictors of response will likely involve monitoring the rapid downregulation of key KMT2A-AFF3 target genes, such as HOXA9 and MEIS1, or the reversal of the pathogenic H3K79 hypermethylation signature. Conversely, understanding mechanisms of resistance is equally urgent. Future studies must investigate potential biomarkers of resistance, such as the acquisition of mutations in the MEN1 gene (which would prevent drug binding) or the compensatory hyperactivation of parallel survival pathways, like the RAS/MAPK or PI3K/AKT signaling cascades.

In conclusion, KMT2A-AFF3 ALL represents a formidable clinical challenge, but one that is now solvable. By moving beyond conventional chemotherapy and striking at the core epigenetic dependencies of the disease, the new generation of targeted therapies offers the first tangible hope of improving survival and fundamentally altering the natural history of this devastating leukemia.

## Figures and Tables

**Table 1:** Key Downstream Signaling Pathways Activated in KMT2A-r Leukemia.

Signaling Pathway	General Role in ALL	Specific Relevance to KMT2A-r/KMT2A-AFF3
Wnt/β-catenin	Regulates self-renewal, proliferation, and cell fate.	Often hyperactivated in KMT2A-r/β-catenin stabilization is crucial for maintaining the “leukemia stem cell” (LSC) properties and contributes to leukemic cell survival [21].
PI3K/AKT/mTOR	Central regulator of cell growth, survival, metabolism, and proliferation.	Frequently activated in ALL; in KMT2A-r, it is a major driver of resistance to chemotherapy, particularly glucocorticoids [22,23]. It also promotes the Warburg effect (aerobic glycolysis) and protein translation via mTORC1.
RAS/MAPK	Transduces signals from growth factor receptors to control cell proliferation and survival.	Co-mutations in RAS pathway genes (e.g., KRAS, NRAS, PTPN11) are very common “second hits” in KMT2A-r ALL, cooperating with the fusion gene to provide a potent proliferative signal [24].
JAK/STAT	Mediates cytokine signaling, crucial for hematopoietic cell proliferation and differentiation.	JAK/STAT signaling, particularly via STAT5, is often activated by KMT2A-r-driven autocrine loops (e.g., upregulation of FLT3 or cytokine receptors), promoting cell cycle progression and survival [19,25]

**Table 2:** Summary of Diagnostic Modalities for KMT2A-AFF3 ALL.

**Methodology**	**Target**	**Purpose/Clinical Utility**	**Limitations**
Flow Cytometry	Cell surface/intracellular proteins (CD markers)	Diagnosis and Classification. Identifies B-lineage (CD19+), pro-B/pre-B stage, and hallmark CD10-negative phenotype. Detects aberrant myeloid markers.	Not specific to KMT2A-AFF3. Cannot distinguish from other KMT2A-r subtypes.
Conventional Cytogenetics	Chromosomes (G-banding)	Initial identification of the t(2;11)(q11.2;q23) translocation.	Low resolution. May miss cryptic or complex translocations [7]. Labor-intensive.
FISH	KMT2A gene locus (11q23)	Rapid Screening. Confirms a KMT2A rearrangement (“break-apart” signal) with high sensitivity.	Does not identify the AFF3 partner. Requires follow-up testing.
RT-PCR	KMT2A-AFF3 chimeric mRNA	Specific Confirmation. Confirms the exact fusion transcript KMT2A-AFF3.	Requires a priori suspicion of AFF3 as the partner to select the correct primers.
NGS (RNA-seq)	Total transcriptome (cDNA)	Gold Standard Identification. Unbiased detection of KMT2A-AFF3 and any other fusions. Identifies precise breakpoints. Simultaneously finds cooperating mutations (e.g., RAS pathway) [24].	Higher cost and longer turnaround time than PCR. Requires bioinformatics expertise.
qPCR/ddPCR	KMT2A-AFF3 DNA/cDNA junction	MRD Monitoring. Ultra-sensitive quantification of residual leukemia cells during and after therapy to guide treatment decisions [27].	Patient-specific assay must be designed and validated after diagnosis. Not a primary diagnostic tool.

**Table 3:** Key Prognostic Factors in KMT2A-AFF3 and KMT2A-rearranged ALL.

Prognostic Factor	Favorable Indicator	Unfavorable Indicator (High-Risk)	Clinical Significance and Relevance to *KMT2A-AFF3*
Primary Genetic Lesion	ETV6-RUNX1, High Hyper diploidy	Presence of KMT2A-AFF3 (or any KMT2A-r)	Defines the primary risk group. The KMT2A-AFF3 fusion is the initiating driver and assigns the patient to high-risk therapy from diagnosis [3,28].
Age at Diagnosis	1–9.99 years	Infancy (<1 year), especially <6 months	A critical independent risk factor. KMT2A-AFF3 often occurs in infants, compounding the genetic risk and leading to a very poor prognosis [26].
WBC at Diagnosis	<50,000/µL (B-ALL)	>50,000/µL (Hyperleukocytosis)	Indicates high tumor burden. KMT2A-AFF3 patients frequently present with hyperleukocytosis, another independent high-risk factor.
Early Treatment Response (MRD)	Rapid clearance (e.g., <0.01% at End of Induction)	Persistent MRD (e.g., >0.1% at End of Induction)	The most powerful prognostic indicator. Assesses in vivo chemosensitivity. Persistent MRD in KMT2A-AFF3 ALL is a strong indication for HSCT [29,30].
Cooperating Mutations	Absence of “second hits”	Presence of RAS pathway mutations (KRAS, NRAS, PTPN11)	Modifies disease biology. KMT2A-r ALL has a high frequency of cooperating RAS mutations, which enhance proliferation and chemoresistance [24].
CNS Status	CNS-1 (No blasts in CSF)	CNS-2 or CNS-3 (Blasts in CSF)	Indicates disease spread. KMT2A-r leukemias, including KMT2A-AFF3, have a high propensity for CNS infiltration, requiring intensive CNS-directed therapy [3].

**Table 4:** Representative Clinical Trials of Menin Inhibitors in KMT2A-r Acute Leukemia.

Drug Name (Code)	Target	Trial Name/ID	Phase	Target Population	Key Reference(s)
Revumenib (SNDX-5613)	Menin-KMT2A interaction	AUGMENT-101 (NCT04065399)	Phase I/II (Pivotal)	Relapsed/Refractory (R/R) KMT2A-r or NPM1-mutant (NPM1m) acute leukemia (ALL & AML); Adult and Pediatric	Issa et al., 2023 [38]; Aldoss et al., 2023 [39]
Ziftomenib (KO-539)	Menin-KMT2A interaction	KOMET-001 (NCT04067336)	Phase I/II	R/R KMT2A-r or NPM1m acute myeloid leukemia (AML); includes KMT2A-r ALL sub-study	Wang et al., 2020 [40] Falkenstein et al., 2022 [41]
Revumenib + Chemo. (e.g., Venetoclax)	Menin-KMT2A interaction and BCL-2	SAVE (NCT05360160)	Phase I/II	R/R AML or Myeloid Leukemia with KMT2A-r, NPM1m, or NUP98-r	Issa et al., 2023 [42]

## Data Availability

No new data were created during this study.
